# A novel splicing mutation *DNAH5* c.13,338 + 5G > C is involved in the pathogenesis of primary ciliary dyskinesia in a family with primary familial brain calcification

**DOI:** 10.1186/s12890-024-03164-w

**Published:** 2024-07-16

**Authors:** Xiu-juan Yao, Qian Chen, Hong-ping Yu, Dan-dan Ruan, Shi-jie Li, Min Wu, Li-sheng Liao, Xin-fu Lin, Zhu-ting Fang, Jie-wei Luo, Bao-song Xie

**Affiliations:** 1 Fujian Provincial Hospital, Shengli Clinical Medical College of Fujian Medical University, no. 134 East Street, Fuzhou, 350001 China; 2 Respiratory department, Fujian Provincial Hospital, Fuzhou, China; 3 Interventional Department, Fujian Provincial Hospital, Fuzhou, China; 4 Department of Hematology, Fujian Provincial Hospital, Fuzhou, China; 5 Pediatrics department, Fujian Provincial Hospital, Fuzhou, China; 6 Department of Traditional Chinese Medicine, Fujian Provincial Hospital, Fuzhou, China

**Keywords:** Primary ciliary dyskinesia, Primary familial brain calcification, *DNAH5*, *MYORG*, Splicing mutation

## Abstract

**Background:**

Primary ciliary dyskinesia (PCD) is an autosomal recessive hereditary disease characterized by recurrent respiratory infections. In clinical manifestations, *DNAH5* (NM_001361.3) is one of the recessive pathogenic genes. Primary familial brain calcification (PFBC) is a neurodegenerative disease characterized by bilateral calcification in the basal ganglia and other brain regions. PFBC can be inherited in an autosomal dominant or recessive manner. A family with PCD caused by a *DNAH5* compound heterozygous variant and PFBC caused by a *MYORG* homozygous variant was analyzed.

**Methods:**

In this study, we recruited three generations of Han families with primary ciliary dyskinesia combined with primary familial brain calcification. Their clinical phenotype data were collected, next-generation sequencing was performed to screen suspected pathogenic mutations in the proband and segregation analysis of families was carried out by Sanger sequencing. The mutant and wild-type plasmids were constructed and transfected into HEK293T cells instantaneously, and splicing patterns were detected by Minigene splicing assay. The structure and function of mutations were analyzed by bioinformatics analysis.

**Results:**

The clinical phenotypes of the proband (II10) and his sister (II8) were bronchiectasis, recurrent pulmonary infection, multiple symmetric calcifications of bilateral globus pallidus and cerebellar dentate nucleus, paranasal sinusitis in the whole group, and electron microscopy of bronchial mucosa showed that the ciliary axoneme was defective. There was also total visceral inversion in II10 but not in II8. A novel splice variant C.13,338 + 5G > C and a frameshift variant C.4314delT (p. Asn1438lysfs *10) were found in the *DNAH5* gene in proband (II10) and II8. c.347_348dupCTGGCCTTCCGC homozygous insertion variation was found in the *MYORG* of the proband. The two pathogenic genes were co-segregated in the family. Minigene showed that *DNAH5* c.13,338 + 5G > C has two abnormal splicing modes: One is that part of the intron bases where the mutation site located is translated, resulting in early translation termination of *DNAH5*; The other is the mutation resulting in the deletion of exon76.

**Conclusions:**

The newly identified *DNAH5* splicing mutation c.13,338 + 5G > C is involved in the pathogenesis of PCD in the family, and forms a compound heterozygote with the pathogenic variant *DNAH5* c.4314delT lead to the pathogenesis of PCD.

**Supplementary Information:**

The online version contains supplementary material available at 10.1186/s12890-024-03164-w.

## Introduction

Primary ciliary dyskinesia (PCD) is a genetically heterogeneous autosomal recessive disease, characterized by abnormal or absence of ciliary movement in various organs. The clinical manifestations of PCD include chronic upper and lower respiratory diseases, left and right lateral abnormalities and infertility [1, 2]. Lung-sinus symptoms are usually the most common manifestation of the condition. Visceral inversion occurs in approximately 50% of patients with PCD [3]. PCD is widely present in all ethnic groups, with no racial or gender preference. PCD is reported to typically affect 1/10,000 people [4]. However, there are some reports of its prevalence in children with recurrent respiratory infections as high as 5% [5]. Most PCD-related genes encode proteins involved in axoneme movement, structure and regulation or cilia assembly and preassembly. Over the past decade, the discovery of new genes has accelerated, with nearly 50 genes are associated with the disease [6, 7]. *DNAH5* mutations are responsible for defective outer dynamin arm (ODA) in PCD patients and the mutations are clustered in five exons (34, 50, 63, 76, and 77) [8]. *DNAH5* is located on chromosome 5p15.2 and contains 79 exons, encoding a heavy chain of the ODA [9]. *DNAH5* is related to the activity of ATPase and microtubule motor protein. Th deficiency of *DNAH5* may cause ODA defects and induce to the appearance of PCD [8].

Primary familial brain calcification (PFBC), is a rare neurodegenerative disease characterized by bilateral calcium deposits in the basal ganglia, which may also involve the cerebellar dentate nucleus, thalamus, subcortical white, cerebellar cortex, and Cerebral cortex. The average age of clinical onset of PFBC is about 40–50 years old, with an estimated prevalence of 2.1 per 1,000 [10]. PFBC follows an autosomal dominant or recessive inheritance. Recessive hereditary PFBC is caused by three genes: *MYORG* [11], *JAM2* [12], and *CMPK2* [13]. *MYORG* is located on chromosome 9p13.3 and contains two exons. *MYORG* is a glycosidase that is mainly located in the nuclear membrane (NE)/ endoplasmic reticulum lumen (ERL) [14].

We found a rare combination of PFBC in a PCD family. In this study, the genetic investigation of this family was conducted to analyze the function of the novel splicing variant of *DNAH5* for reference by clinicians.

## Methods

### Research subjects

A Chinese family with PCD and PFBC from Fujian, China; the family was comprised of 9 members in 3 generations, including 3 males and 6 females. This study was carried out with the approval of the Ethics Committee of Fujian Provincial Hospital. Recruited family members were informed and voluntarily signed the informed consent form before the clinical investigation. There is no single gold standard diagnostic test for PCD [15]. According to the diagnostic recommendations based on the American Thoracic Society guidelines [16], at least two out of four key clinical features of PCD: a full-term newborn with unexplained respiratory distress, perennial daily cough and nasal congestion begin in 6 months of age, and laterality defects. On this basis, PCD can be diagnosed by the presence of 1 of the followings: Low nasal nitric oxide levels were repeatedly verified and cystic fibrosis was excluded, double allele pathogenic variants in PCD-related genes, ciliary ultrastructural defects were observed by electron microscope in single or non-pathogenic variant of PCD genes. The diagnosis of PFBC is based on the display of bilateral basal ganglia calcification on brain imaging (CT scan) and the exclusion of secondary causes of calcium deposition in the brain [17].

### Clinical phenotype

The history and clinical data, computed tomography (CT), magnetic resonance imaging (MRI), color doppler ultrasound, laboratory index, nasal nitric oxide detection, electron microscopic examination of cilia ultrastructure, bronchoscope examination of the family members were collected, and the clinical phenotypic correlation was analyzed.

### DNA extraction

Peripheral blood of the proband and family members were collected with ethylenediaminetetraacetic acid anticoagulant tube, and genomic DNA was extracted according to the kit’s instructions (QIAamp DNA Blood Mini Kit, GER). The DNA concentration and purity were measured using Nanodrop 1000 (Nanodrop Technologies, USA).

### Candidate gene mapping and mutation screening strategy

IDT’s liquid phase chip capture system technology was used to construct genomic DNA library, and the exon regions (39 Mbp) containing 19,989 genes associated with genetic diseases were captured, and the PE150 was sequenced by Illumina Novaseq-6000 platform. The target genes involved in single-gene genetic diseases included *DNAH5*, *HYDIN*, etc. Based on the BAM results compared to genomic reference sequences, software such as SAMtools was used to find and analyze SNVs and indels in the sequencing data. According to the ACMG guidelines, the variants were screened and annotated using the human genome information from OMIM, HGMD, ClinVar and other databases. The sequenced reads was compared with the reference genome sequence (GRCh37/hg19), and learned the important information of these variants, such as gene information, mutation type, frequency of 1000G and ESP6500, PolymorpHism Phenotyping (PolyPhen-2, http://genetics.bwh.harvard.edu/ppH2/), Sorting Intolerant from Tolerant (SIFT, http://sift.jcvi.org/) and Mutation Taster(http://mutationtaster.org/) were used to predict the mutation pathogenicity. Premier 5 software was used to design amplification primers for the sequence location of the target mutation site, and the target region was amplified. The amplified fragment of the target sequence of *DNAH5* (NM_001369.3) c. 13,338 + 5G > C was 265 bp, and The primers used were F: GTCCGCAGCACCCTCACT; R: AAAACTTCCTTCCCTACCTATGG. The annealing temperature was 62 ℃.

### Construction and verification of plasmid

The *DNAH5* wild type and c. 13,338 + 5G > C mutant were constructed into pCAS2. Exon sites were extended 150 bp intron sequences on both sides. If the exon sequence was less than 150 bp, the exon sequence could be extended to both ends, KpnI and BamHI were selected as the double restriction sites. *DNAH5* (WT), *DNAH5* (c.13,338 + 5G > C) gene cloning and related PCR primers synthesis were performed by Genecreate Bioengineering Co. Ltd (Wuhan, China).

### Cell transfection

HEK293T cells were collected by trypsin digestion. The cells were incubated at 37 ℃ incubator with 5% CO2 for 24 h, transient transfection was started after the cells were fully adhered. TurboFect-DNA Mix was prepared according to the instructions of TurboFect. 4 µg pCAS2 and 8 µL TurboFect were added to 400 µL Opti-Medium, mixed gently and incubated at room temperature for 15 min. TurboFect-DNA Mix was added to the petri dish. After transfection overnight, the complete medium was changed and the cells were cultured for 24 h.

### Reverse transcription PCR (RT-PCR)

Total RNA was isolated from HEK293T cells using TRIPURE ISOLATION REAGENT kit (11,667,165,001, Roche, Switzerland). Reverse transcription was performed according to the following reaction system: 2.5 mM dNTP Mix, 4µL; Primer Mix, 2µL; RNA Template, 7µL; 5 × RT Buffer, 4 µL; 0.1 M DTT, 2µL; 200 U/µL HiFiScript (CW2020M, CWBIO, Beijing, CHN), 1µL; RNase-Free Water to a final volume of 20µL. Mix the solution with vortex shock and centrifuge for a short time. The samples were incubate at 42 ℃ for 50 min and at 85 ℃ for 5 min. PCR amplification was performed using HS Premix system, and the PCR products were identified by agarose, and recovered for sequencing.

## Results

### Clinical phenotype

**Fig. 1:**
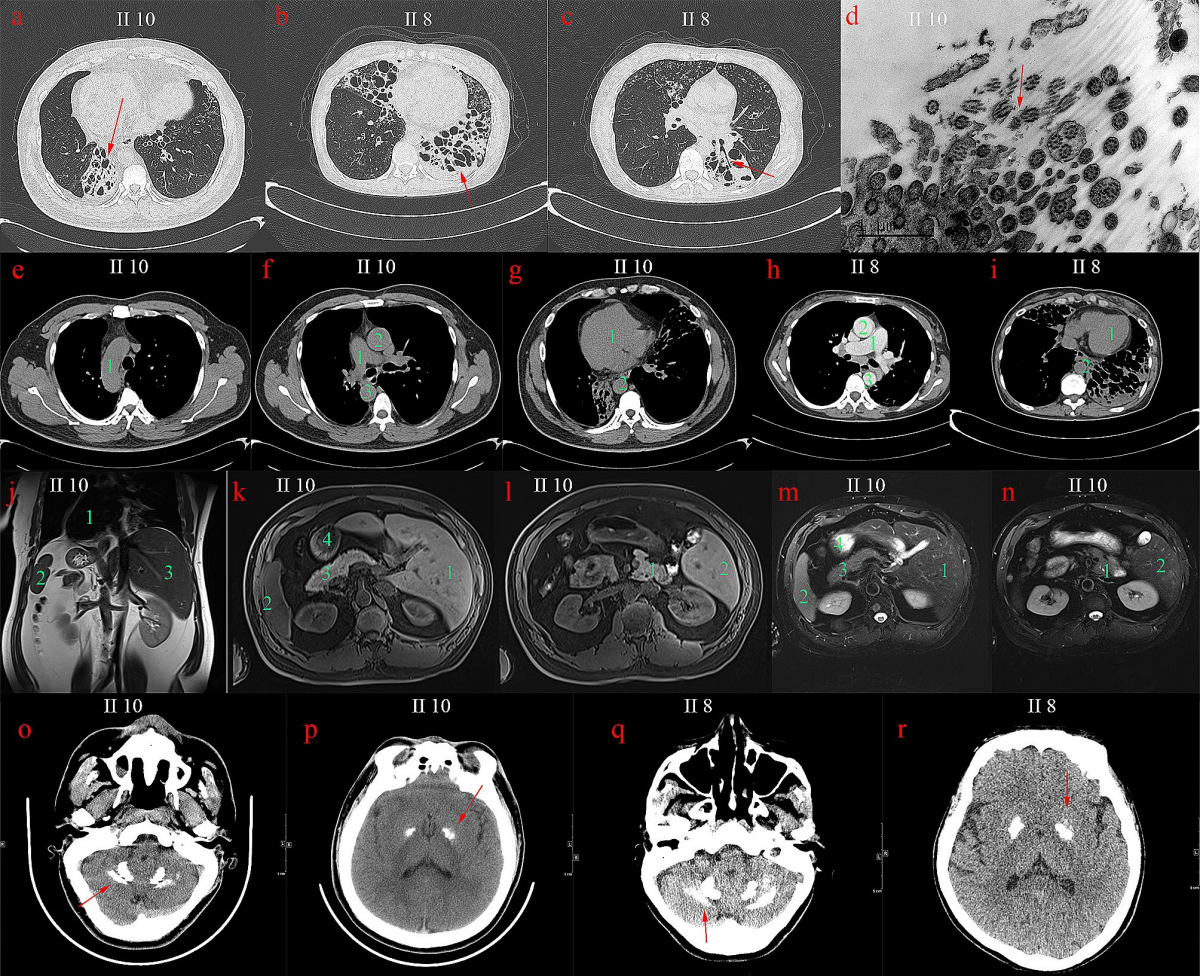
(**a**) Chest CT images of proband II10: the cystic and columnar expansion of the both lower lung bronchi. (**b**) Chest CT images of II8: the cystic dilatation of left lower lung and right middle lung bronchi. (**c**) bronchial cystic dilation and mucus filling in the left lower lung, tree-in-bud pattern was seen in the middle lobe of the right lung. (**d**) Bronchial mucosa of II10 was observed by electron microscopy, the ultrastructure of cilia was disordered, some microtubules were arranged in disorder, surrounding microtubules were reduced. (**e**) The right aortic arch of II10. (**f**) The right pulmonary aorta (1), right ascending aorta (2) and descending aorta (3) of II10. (**g**) The dextrocardia of II10. (**h**) The normal positions of pulmonary trunk, ascending aorta and descending aorta of II8. (**i**) The normal position of heart of II8; (**j-n**) Abdominal CT images of II10: the reversal of the position of the heart, liver, spleen, pancreas, and stomach. (**o-r**) Brain CT images of II10 and II8. **o**, **q**, Bilateral calcifications observed in dentate nucleus; **p**, **r**, bilateral and symmetrical calcifications observed in globus pallidus

**Fig. 2:**
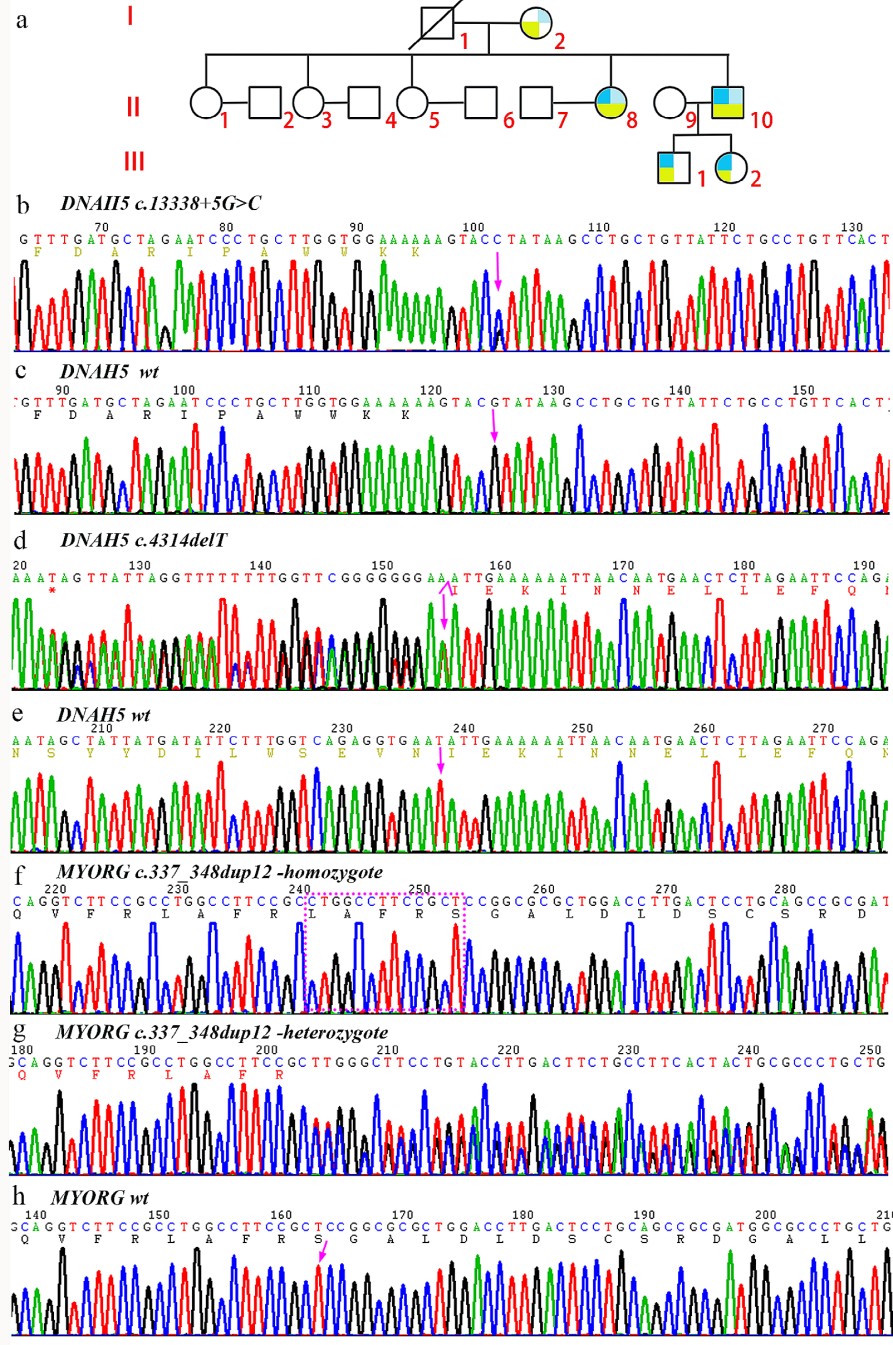
(**a**) Family map: dark blue indicates c.13,338 + 5G > C variant carrying *DNAH5* gene (NM_001369.2); light blue indicates c.4314delT (p.Asn1438Lysfs*10) variant carrying *DNAH5* gene; light green indicates c.337_348dup12 (p.Arg116_Ser117insLeu AlaPheArg) variant carrying *MYORG* gene (NM_020702.5); (**b-h**) Sanger sequence diagram: **b**, the proband carried the *DNAH5* variant c.13,338 + 5G > C; **c**, wild-type at DNAH5 c.13,338 + 5; **d**, the proband carried the *DNAH5* variant c.4314delT (p.Asn1438Lysfs*10); **e**, wild-type of DNAH5 c.4314; **f**, *MYORG* p.Arg116_Ser117insLeuAlaPheArg homozygous mutation; **g**, *MYORG* p.Arg116_Ser117insLeu Ala Phe Arg heterozygous mutation; **h**, wild-type of *MYORG*

Two of the nine members were diagnosed with PCD combined with PFBC (II 8, II 10). The proband (II 10), 39 years old, had a history of repeated cough, sputum and nasal congestion for more than 30 years, accompanied with intermittent hemoptysis, and had a previous history of infertility and otitis media. He had a son and a daughter through assisted reproduction. Physical examination revealed moist crackles in both lungs, no pestle finger. Chest CT showed multiple bronchiectasis (Fig. 1a) in both lungs, and the nitric oxide in the nasal exhalation was determined as 22ppb. Bronchoscopy showed a moderate amount of thick secretions in each Lobular segments of the bilateral bronchial lumen and luminal purulent secretions were aspirated from the left lower lobe and right middle ducts during negative pressure suction. Pseudomonas aeruginosa was detected by bronchial secretion culture. The ultrastructural observation of respiratory cilia revealed abnormalities in the ciliary axoneme, encompassing defects in the inner and outer dynamic arms (IDAs/ODAs) (Fig. 1d). Chest CT and MRI of the chest and abdomen revealed visceral inversion (Fig. 1e-g, j-n). Brain CT showed multiple symmetrical calcifications of bilateral globus pallidus and cerebellar dentate nucleus (Fig. 1o, p). Paranasal sinus CT showed paranasal sinusitis and bilateral middle ear mastoiditis. Cardiac color doppler ultrasound showed mirror dextrocardia with no abnormality in cardiac structure and function. The sister (II 8) of the proband had cough, purulent sputum and nasal congestion since childhood, had a previous history of massive hemoptysis once. She had a son. Chest CT showed multiple bronchiectasis (Fig. 1b, c) in both lungs without visceral inversion (Fig. 1h, i). Paranasal sinus CT revealed chronic paranasal sinusitis and bilateral middle ear mastoiditis. Brain CT showed multiple symmetrical calcification in bilateral basal ganglia, globus pallidus and cerebellar dentate nucleus (Fig. 1q, r). The other members of the family were investigated for phenotype and variant carriage, and the pedigree map was drawn (Fig. 2a; Table 1). The father of the proband died at the age of 59 due to cutaneous malignancy, and no bronchiectasis, sinusitis, visceral inversion and brain calcification were found during his life.

**Table 1:** The clinical presentation of the studied family members

ID	Sex	Age of onset/Age at the time of investigation (year)	Bronchiectasis	Paranasal sinusitis	Visceral inversion	Cerebral calcification	Infertility	Otitis media	Outcome
I1	M	- / 59	-	-	-	N/A	-	-	Death (cutaneous malignancy)
I2	F	- / 69	-	-	-	-	-	-	Alive
II1	F	- / 47	-	-	-	-	-	-	Follow-up
II3	F	- / 45	-	-	-	-	-	-	Follow-up
II5	F	- / 42	-	-	-	-	-	-	Follow-up
II8	F	3 / 42	+	+	-	+	-	+	Under treatment
II10	M	4 / 39	+	+	+	+	+	+	Under treatment
III1	M	- / 4	-	-	-	-	-	-	Follow-up
III2	F	- / 7	-	-	-	-	-	-	Follow-up

Note: N/A, not available

### Screening for gene mutations associated with PCD combined with PFBC

Through next-generation sequencing (NGS) and exon capture technology, the proband (II10) was found to have a *DNAH5* (NM_001361.3) splicing variation C.13,338 + 5G > C (Fig. 2b, c). We also found a base T deletion in exon 27 of another *DNAH5* allele, resulting in a frameshift of 10 amino acids, named c.4314delT (p. Asn1438Lysfs * 10) (Fig. 2d, e). The frameshift variant translates a novel 10 amino acid peptide before a stop codon, resulting in a new 1448 amino acid protein. In addition, the c.337_348dupCTGGCCTTCCGC mutation (Fig. 2f-h) was found in exon 2 of *MYORG* (NM_020702.5). The insertion variant inserts 12 bases between the coding regions 347 and 348, resulting in the insertion of four amino acids Leu, Ala, Phe and Arg between the residue 116 and the residue 117. c.337_348dup CTGGCCTTCCGC mutation has been reported as pathogenic by ClinVar database. Sanger sequencing revealed that the *MYORG* c.347_348dup12 heterozygous variation was found in family members I2, III1 and III2, while the *MYORG* c.347_348dup12 homozygous variation was detected in II8. II8 carried a compound heterozygous variant of *DNAH*5 c. 13,338 + 5G > C and *DNAH5* c.4314delT, III1 and III2 carried a heterozygous variant of *DNAH5* c. 13,338 + 5G > C (Fig. 2a). Cosegregation between genotypes and phenotypes associated with PCD and PFBC was observed in this family.

### Cloning of DNAH5 mutants

The cloning of *DNAH5* WT and *DNAH5* (c.13,338 + 5G > C) and eukaryotic expression vectors of pCAS2-*DNAH5* c. 13,338 and pCAS2-*DNAH5* c.13,338 + 5G-C were successfully constructed. The fragments of *DNAH5* WT and *DNAH5* (c. 13,338 + 5G > C) digested by KpnI and BamHI were about 150 bp, which was consistent with the design. The constructed vectors were verified by Sanger sequencing and transiently transfected into HEK293T cells.

### Minigene splicing assay

**Fig. 3:**
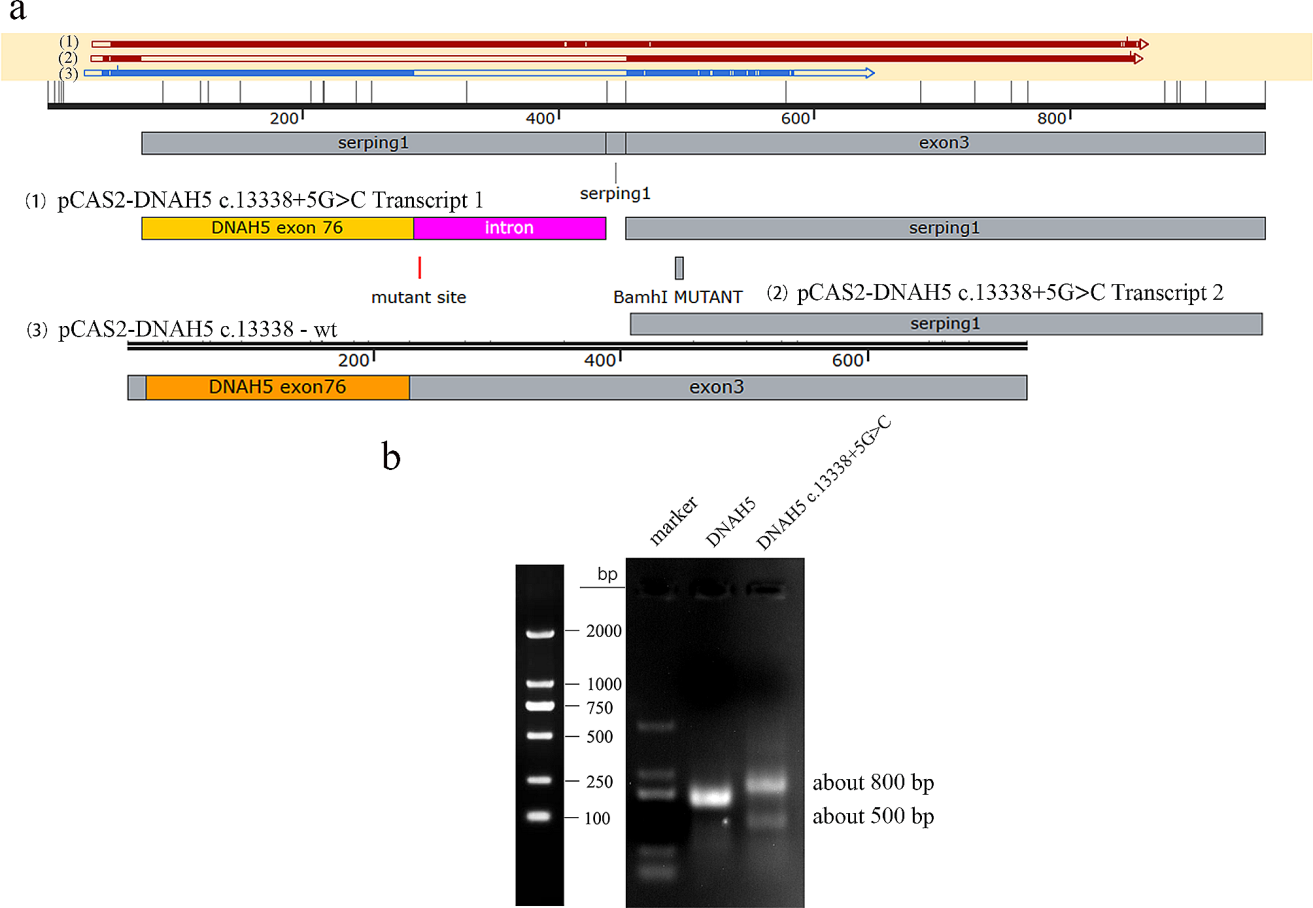
Minigene splicing assay The splicing pattern of *DNAH5* WT was consistent with the report of NCBI, while *DNAH5* mutation c.13,338 + 5G > C showed two types of splicing, with the target band of about 800 bp and 500 bp. The splicing form of 800 bp band was that some bases of the intron where the mutation site is located were translated; The splicing form of 500 bp band was the deletion of exon 76 where the c.13,338 is located. (**a**) A diagram of splicing: (1), (2) and (3) represent three forms of splicing respectively. (**b**) Agarose gel results of splicing modes of DNAH5 mutation c.13,338 + 5G > C and wild-type; full-length gels are presented in Supplementary Figure

**Fig. 4:**
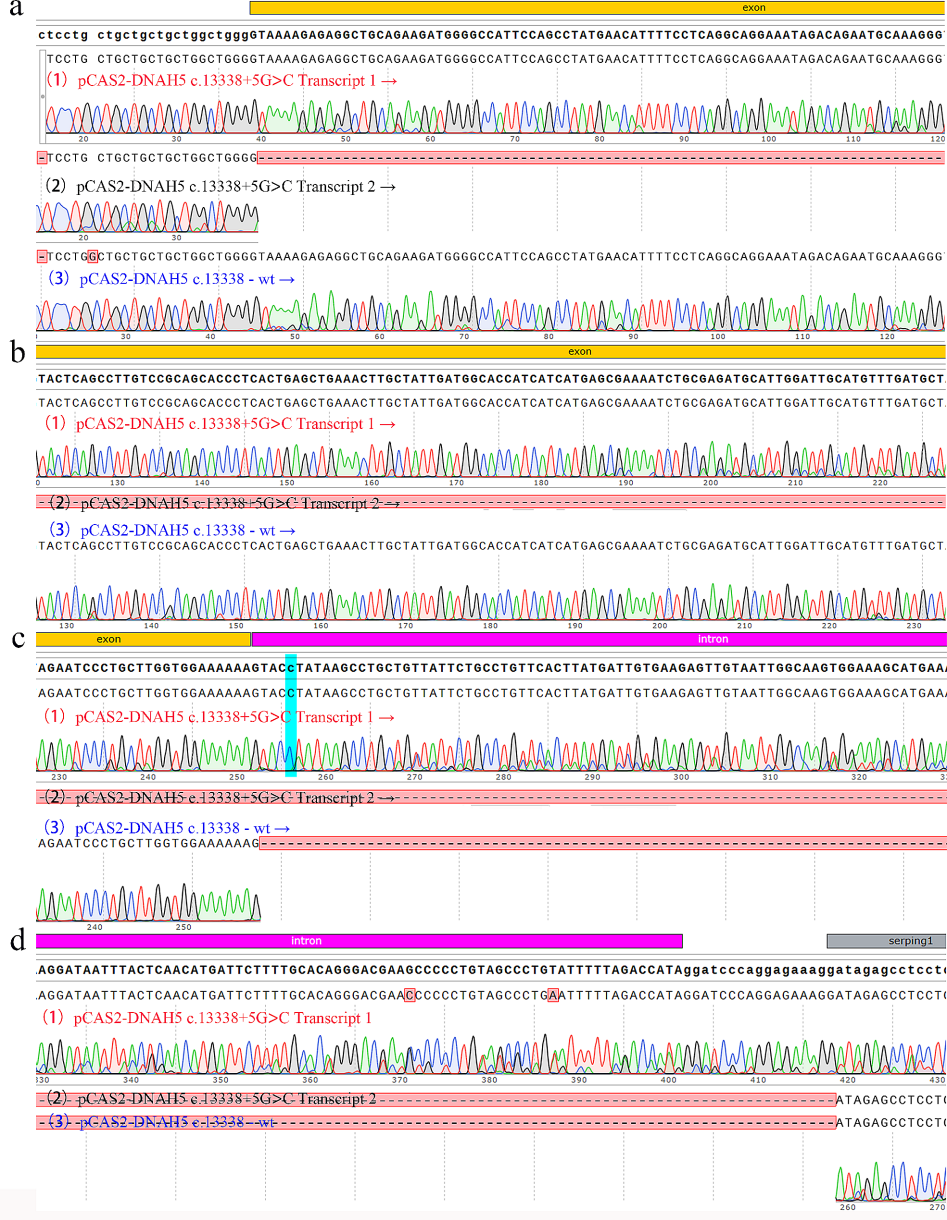
RNA sequencing results after splicing were detected by Minigene splicing assay. (**a-d**) sequencing fragments of different regions, respectively. (1) and (2), Sequencing results of 800 bp and 500 bp spliced after transfection with pCAS2- *DNAH5* c.13,338 + 5G> C. (3), Sequencing results of splicing after transfection with pCAS2-*DNAH5* c.13,338-WT

The splicing modes of *DNAH5* WT and *DNAH5* c.13,338 + 5G > C were detected. The splicing pattern of wild-type was consistent with the report of NCBI, while the mutation showed two types of splicing. The target bands identified by Agarose gel were about 800 bp and 500 bp (Fig. 3b). The splicing form of 800 bp band was that some bases of the intron where the mutation site is located were translated, leading to the early termination of DNAH5 translation, and the splicing form of 500 bp band was the deletion of exon 76 where the c.13,338 is located (Figs. 3a and 4).

### Bioinformatics analysis

**Fig. 5:**
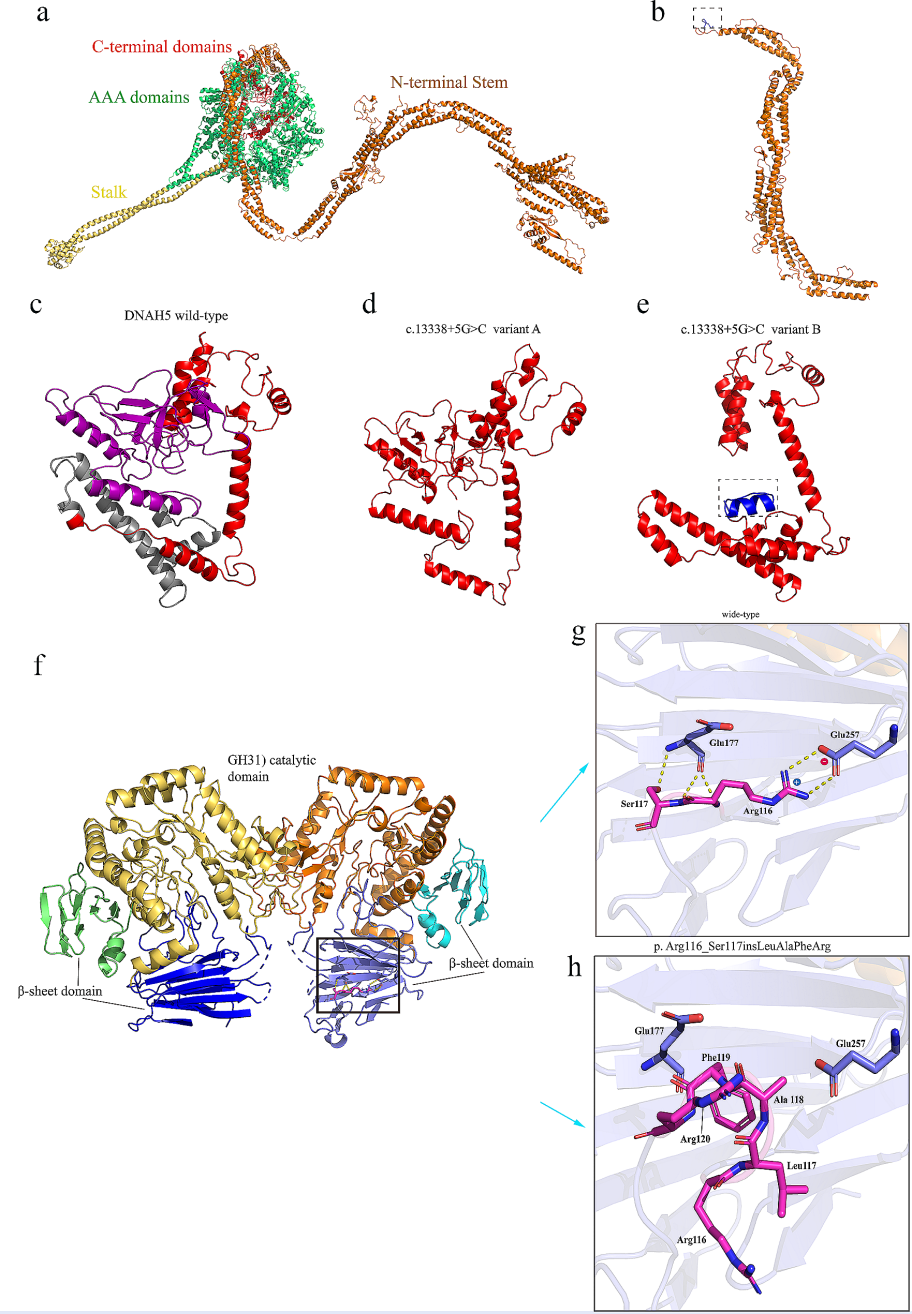
Swiss-model (https://swissmodel.expasy.org/), Phyre2 (http://www.sbg.bio.ic.ac.uk/~phyre2/html/page.cgi?id=index) and I-TASSER (https://zhanggroup.org/I-TASSER/) were used to predict the three-dimensional structure of dynamin axonal heavy chain 5 *DNAH5* WT, *DNAH5* mutants, myogenesis-regulated glycosidase *MYORG* WT and *MYORG* mutants and shown in Pymol. (**a**) Three-dimensional structure map of *DNAH5* wild-type. (**b**) Three-dimensional structure map of *DNAH5* c.4314delT (p.Asn1438Lysfs*10). The blue region in the dashed box indicates the additional 10 amino acids caused by frameshift. (**c**) The c-terminal globular fragment of *DNAH5* WT: the gray area shows the deletion of exon 76 in the c.13,338 + 5G > C mutant, resulting in the deletion of 71 amino acids (c. 13,338 + 5G > C mutant A); the purple region indicates translation terminated after 18 amino acids have been translated from the intron where the mutation site is located, resulting in the deletion of 178 amino acids (c.13,338 + 5G > C mutant B). (**d**) Three-dimensional structure map of c. 13,338 + 5G > C variant A. (**e**) The dashed box in c. 13,338 + 5G > C mutant B indicates an additional 18 amino acids caused by abnormal splicing. (**f-h**) Prediction and comparison of the three-dimensional structure of the *MYORG* wild-type and the variant p.Arg116_Ser117insLeuAlaPheArg: residues 116, 117 and interacting residues are represented as sticks and labeled. Electrostatic interactions and hydrogen bonds are represented by dotted lines; positively and negatively charged residues are marked with a blue (+) and red (-) symbol, respectively

To assess the effect of mutations on protein structure, the computer models of *DNAH5* WT, *DNAH5* c.13,338 + 5G > C and *DNAH5* c.4314delT (p. Asn1438Lysfs), *MYORG* WT and *MYORG* c.337_348dupCTGGCCTTCCGC (p. Arg116_Ser117insLeuAlaPheArg) were established by swiss model, Phyre2 and I-TASSER (Fig. 5). The three-dimensional structure showed that the frameshift region of the Asn1438Lysfs mutant caused the deletion of 3187 amino acids. The addition of 10 amino acids to the *DNAH5* c.4314delT (p. Asn1438Lysfs) mutant resulted in a frameshift mutation. There are two splicing modes in *DNAH5* c. 13,338 + 5G > C mutant. One form of splicing is translation terminated after 18 amino acids have been translated from the intron where the mutation site is located, resulting in early translation termination of *DNAH5* and deletion of 178 amino acids in *DNAH5* WT. Another splicing form is the deletion of exon 76 where c.13,338 is located, causing the deletion of 71 amino acids encoded by exon 76 in *DNAH5* WT. In addition, In the *MYORG* WT, p. Arg116 forms a salt-bridge with p.Glu257 and p.Ser117 forms a hydrogen bond with p.Glu177. In the P. Arg116_Ser117insLeuAlaPheArg variant, the above interactions disappeared, resulting in the loss of connectivity and stability of the variant.

## Discussion

PCD is a motile ciliated hereditary disease caused by decreased mucosal ciliary clearance capacity, resulting in recurrent respiratory infections [18]. Cilia are microtubule tissue structures protruding from the cell surface, which is composed of more than 200 kinds of proteins. These proteins form nine peripheral microtubule doublets composed of A and B microtubules, which are arranged around a pair of central microtubules (a typical “9 + 2” arranged microtubule doublet) [19, 20]. The heavy, intermediate and light chains of dynamin are assembled into multiprotein complexes, forming inner dynamic arm (IDA) and outer dynamic arm (ODA). IDA and ODA are attached to A microtubules of peripheral axial filaments, promoting microtubule sliding through ATPase activity [21]. Therefore, cilia lacking ODA may appear stiff or ineffective movements.

Human respiratory cilia contain at least two types of ODA: ODA type 1 (*DNAH5* positive and *DNAH9* negative), mainly distributed in the proximal ciliary axoneme. ODA type 2 (*DNAH5* and *DNAH9* positive), mainly distributed in the distal ciliary axoneme. Hornef et al [8]. performed haplotype analysis and sequencing of 109 PCD families and found that in patients with IVS76 + 5G > A and IVS75-2AT compound heterozygous mutations, the mutant *DNAH5* was absent from distal ciliated axoneme, but retained in the proximal ciliated axoneme. *DNAH5* splicing mutation C.13,338 + 5G > C has the same mutation site as *DNAH5* IVS76 + 5G > A. This indicates that the C-terminal region of *DNAH5* is crucial for the assembly of ODA Type 2. The results of minigene detection and bioinformatics prediction revealed that the c. 13,338 + 5G > C splice mutation would generate two types of abnormal splicing, interfere with OAD type 2 assembly and lead to the defects of distal ciliated axoneme.

The dynein heavy chain consists of an N-terminal stem, six AAA domains (AAA1-6) and C-terminal globular fragments assembled into heptagonal rings [22, 23]. The N-terminal stem can bind cargo and interact with other dynein components. Each AAA domain contains a ATPase site (4 conserved ATPase sites and 2 non-conservative ATPase sites). Probably the ATPase located in the AAA1 domain actually hydrolyzes ATP, while others play a regulatory role. The tip of a stem-like structure between AAA4 and AAA5 has a microtubule binding site.

Loss-of-function variants in *DNAH5*, which encodes the heavy chain of ODA, are known to be pathogenic (Table 2). These functional deletion mutations lead to ODA defects, resulting in cilia immobility or severe motor changes (such as stiff movements, low amplitudes) and the onset of PCD [8, 24]. In this study, the c.4314delT (p.Asn1438Lysfs*10) *DNAH5* frameshift mutation led to nonsense-mediated mRNA degradation (NMD) or the production of truncated proteins due to premature termination of translation. The truncated protein lost all AAA domains and microtubule-binding sites, interfering with the assembly of ODA. According to the ACMG standard, the mutation was determined to be pathogenic.

**Table 2:** Information on *DNAH5* gene mutations in the proband and some reported mutations

RefSeq	Base change	Amino acid change	Exon	ACMG	Prediction of protein function	Reference
NM_001369.3	c.13,338 + 5G > C	splicing-mut	76	PP1 + PP3 + PM2	Uncertain	This report
NM_001369.3	c.4314delT	p.Asn1438Ly sfs *10	27	PSC1 + PM2 + PP4	Pathogenic	This report
NM_001369.3	[IVS76 + 5G > A]	splicing-mut	76	/	Pathogenic	Hornef et al. PMID: 16,627,867
NM_001369.3	c.8440_8447delGAACCAAA	p.Glu2814fs*1	50	/	Pathogenic	Hornef et al. PMID: 16,627,867
NM_001369.3	c.4361G > A	p.Arg1454Gln	28	/	Pathogenic	Olbrich et al. PMID: 11,788,826
NM_001369.3	c.8910 + 8911delAT→insG	p.Ser2970Leufs*7	53	/	Pathogenic	Olbrich et al. PMID: 11,788,826
NM_001369.3	c.7897_7902delAGAG	p.Glu2633Alafs*18	47	/	Pathogenic	Raidt et al. PMID: 25,186,273
NM_001369.3	C. 10815delT	p.Pro3606Hisfs*22	64	/	Pathogenic	Raidt et al. PMID: 25,186,273

Note: RefSeq, NCBI reference sequence; ACMG, American college of medical genetics and genomics

The gene *MYORG* is the genetic factor of autosomal recessive PFBC. *MYORG* homozygous knockout (KO) mice developed the calcification of bilateral thalamic at the age of 9 months [11]. *MYORG* knockdown zebrafish developed brain calcification around 5 dpf, and the brain calcification phenotype could be alleviated by *MYORG* cDNA compensation [25]. *MYORG* is a type II transmembrane protein. The N-terminal 56-amino acid fragment faces the nucleoplasm followed by a transmembrane domain (residue 57–78). The C-terminal domain faces the plasma membrane and endoplasmic reticulum lumen, including two β-sheet domains (residues 79–287,634–714) and a region homologous to family glycoside hydrolase 31 (GH31, residue 288–633) [14, 26, 27]. The GH31 domain of *MYORG* has six N-glycosylation sites: N240, N250, N346, N372, N398 and N511, and could selectively bind α-galactosides and its substrate Gal-α1,4-Glc [26].

II10 and II8 were both insertional mutant homozygote c.337_348dupCTGGCCTTCCGC, which was consistent with the previous reports [11, 28]. The gene *MYORG* is specifically expressed in the endoplasmic reticulum of astrocytes and regulates protein glycosylation of this region [11]. An autopsy study showed that the calcification deposits found in brain were composed of a mixture of glycoproteins, mucopolysaccharides, calcium salts and irons [29]. Bioinformatic analysis of the p.Arg116_Ser117insLeuAlaPheArg variant suggested that this insertion mutation affected the salt-bridge between Arg116-Glu257 and the hydrogen bond between Ser117-Glu177, causing a decrease in protein stability. In addition, *MYORG* variants may cause the dysfunction of astrocytes, which may affect the normal function of neurovascular units (NVU), leading to the formation of calcified nodules [11].

The patients in this family suffered from both PCD and PFBC, which seemed to be an occasional and rare phenomenon as these two gene loci were not adjacent and the characteristics of the diseases were different. The phenotype of PCD is heterogeneous, the main clinical phenotypes include chronic sinusitis and persistent wet cough, otitis media, bronchiectasis and infertility. The prevalence of PCD in adults with bronchiectasis is estimated to be 1–13% [30]. The visceral inversion associated with PCD is commonly referred to as “Kartagener syndrome”. In this study, both II10 and II8 were compound heterozygous carriers of the DNAH5 c.13,338 + 5G > C variant and the DNAH5 c.4314delT variant. Surprisingly, situs inversus was found in II10, but not in II8. In fact, only about 50% of patients with PCD exhibit mirror reversal of visceral organs, attributed to the dysfunction of embryonic left-right organizer (LRO) cilia, leading to the random left-right orientation of organs [31]. LRO is a temporary embryonic structure capable of establishing proper left-right patterns. Some researchers believe that DNAH5 is a component of motile LRO monocilia, facilitating their movement [32]. The motility of LRO monocilia is a crucial element in generating leftward nodal flow, leading to the correct conduction of the Nodal signaling cascade [33]. The immotility of LRO cilia causes the absence of leftward nodal flow, resulting in Nodal signaling cascades being only occasionally induced on one side of the LRO [32, 33]. The Nodal signaling cascade is a key factor in the normal development of the left-right body axes. Therefore, asymmetric expression of Nodal and related pathway components would lead to the development of either situs solitus or situs inversus [34]. The dyskinesia of the ependymal cilia of the ventricles may promote the accumulation of cerebrospinal fluid in the brain, leading to hydrocephalus [35]. The proband had typical manifestations of PCD such as Kartagener syndrome and infertility, while the sister of the proband did not have these phenotypes, and neither of them showed signs of hydrocephalus.

The main neuropathological feature of PFBC is the existence of calcified nodules along the capillaries at the neurovascular units. The dysfunction of neurovascular units triggered by the formation of calcified nodules is the main mechanism leading to neurodegeneration [36]. The clinical manifestations of PFBC patients include dyskinesia, cognitive impairment, psychosis and cerebellar signs, but may also be asymptomatic [37]. The pathogenic variants of PFBC related genes are 100% penetrance in radiology, but the symptomatic penetrance is reduced, which may be related to specific potential mutated genes [36]. The imaging examination of PFBC patients shows high-density calcifications in the bilateral basal ganglia, with varying degrees of involvement in other brain regions, especially the cerebellar dentate nucleus, thalamus, subcortical white, and cerebral cortex [36]. Two patients (II10, II8) in this family showed symmetrical cerebral calcification in the globus pallidus and cerebellar dentate nucleus. Except for slight dizziness of the sister of the proband, there were no other related symptoms.

## Conclusion

Through clinical characterization and a series of functional studies in a family with PCD and PFBC, a novel splicing variant *DNAH5* c.13,338 + 5G > C was identified, which had two splicing modes, causing ODA assembly disorders and ciliary axonal defects. This variant formed a complex heterozygote with the pathogenic variant *DNAH5* c.4314delT, leading to the related clinical phenotype of PCD patients.

### Electronic supplementary material

Below is the link to the electronic supplementary material.

Supplementary Material 1

## Data Availability

All data generated or analyzed during this study are included in this published article and ClinVar database (https://www.ncbi.nlm.nih.gov/clinvar/variation/2502279/).

## Abbreviations

PCD: Primary ciliary dyskinesia
PFBC: Primary familial brain calcification
ODA: Outer dynamin arm
IDA: Inner dynamic arm
NE: Nuclear membrane
ERL: Endoplasmic reticulum lumen
NGS: Next-generation sequencing
NVU: Neurovascular units
